# Precise Point Positioning Using World’s First Dual-Frequency GPS/GALILEO Smartphone

**DOI:** 10.3390/s19112593

**Published:** 2019-06-06

**Authors:** Abdelsatar Elmezayen, Ahmed El-Rabbany

**Affiliations:** Department of Civil Engineering, Ryerson University, Toronto M5B 2K3, Canada; rabbany@ryerson.ca

**Keywords:** Xiaomi mi 8, PPP, DGNSS, NAVCAST

## Abstract

The release of the world’s first dual-frequency GPS/Galileo smartphone, Xiaomi mi 8, in 2018 provides an opportunity for high-precision positioning using ultra low-cost sensors. In this research, the GNSS precise point positioning (PPP) accuracy of the Xiaomi mi 8 smartphone is tested in post-processing and real-time modes. Raw dual-frequency observations are collected over two different time windows from both of the Xiaomi mi 8 smartphone and a Trimble R9 geodetic-quality GNSS receiver using a short baseline, due to the lack of a nearby reference station to the observation site. The data sets are first processed in differential modes using Trimble business center (TBC) software in order to provide the reference positioning solution for both of the geodetic receiver and the smartphone. An in-house PPP software is then used to process the collected data in both of post-processing and real-time modes. Precise ephemeris obtained from the multi-GNSS experiment (MGEX) is used for post-processing PPP, while the new NAVCAST real-time GNSS service, Germany, is used for real-time PPP. Additionally, the real-time PPP solution is assessed in both of static and kinematic modes. It is shown that the dual-frequency GNSS smartphone is capable of achieving decimeter-level positioning accuracy, in both of post-processing and real-time PPP modes, respectively. Meter-level positioning accuracy is achieved in the kinematic mode.

## 1. Introduction

Precise point positioning (PPP) is a popular global navigation satellite system (GNSS) positioning technique, due to its ability to provide centimeter to decimeter positioning accuracy using a standalone GNSS receiver [1]. Unfortunately, this positioning accuracy level can only be achieved using a costly geodetic-quality GNSS receiver and antenna. Single frequency (SF) PPP has recently become an interesting research topic due to the relatively low cost of SF GNSS receivers and their usage for several market applications. However, the resulting positioning accuracy of SF-PPP is limited to a few decimeters [2]. In May 2016, Google announced the development of an application that makes the raw GNSS measurements available to Android-based smartphone users [3]. The release of such an application software has opened the way for the development of precise positioning techniques using ultra-low-cost sensors. SF GNSS smartphones were tested in rapid-static mode [4] and in network real-time kinematic (NRTK) mode [5]. GPS SF observations from a Nexus 9 smartphone were processed in carrier-phase-based differential mode with respect to continuous operating reference stations (CORS) with baselines ranging from 10 m to 8 km [4]. Decimeter-level position accuracy was achieved after processing of 15 min of data from the smartphone. SF GPS/GLONASS observations from a Samsung Galaxy S8+ and Huawei P10 plus smartphones were also tested and processed in NRTK positioning mode using virtual reference station (VRS) corrections from a CORS network with an inter-station distance of 50 km [5]. Each smartphone’s NRTK position precision was at the centimeter-level, but the true solution accuracy was at the meter-level due in part to the unknown position of the smartphone’s embedded antenna. The main challenge in applying rapid-static and NRTK techniques is the need for either a base station in the vicinity of the surveying area, which is relatively expensive, or a VRS, which is not available in remote regions. To overcome this limitation, the smartphone’s SF GNSS pseudorange and carrier-phase measurements were processed in PPP mode [6,7]. GPS-only pseudorange and carrier-phase measurements from a Nexus 9 smartphone along with precise ephemeris from the international GNSS service (IGS) were used for PPP processing [7]. The global ionospheric model (GIM) was used to account for the ionospheric delay. The obtained smartphone’s PPP solution precision was 25, 28, and 51 cm in East, North and Up directions, respectively. Moreover, the accuracy of the resulted PPP solution from the used SF smartphone could not be evaluated due to the lack of an exact reference solution.

In 2018, Xiaomi mobile brand released the world’s first dual-frequency GNSS smartphone, Xiaomi mi 8. This smartphone is fitted with Broadcom’s BCM47755 chip, which supports L1/L5 frequencies for GPS and E1/E5 frequencies for Galileo in addition to L1 frequency for GLONASS and BeiDou [8]. The availability of pseudorange and carrier phase measurements on L1/E1 and L5/E5 frequencies allows the user to linearly combine the observations to essentially remove the effect of ionospheric delay. This in turn leads to an enhanced PPP positioning solution. This smartphone is also fitted with several sensors such as accelerometers, gyroscopes, a compass, camera, and barometer, which enable it to produce a variety of georeferenced data sets critical for future smart cities [9], road surface monitoring [10], and for low-cost mobile mapping systems [11]. Real-time PPP can also be obtained by taking the advantage of the real-time precise ephemeris service offered, for example, by the IGS [12].

In this research, the performance of Xiaomi mi 8 smartphone is tested in post-processing PPP and real-time PPP modes using GPS/Galileo observations. The real-time positioning performance of the smartphone is assessed in both of static and kinematic modes. In order to provide the reference positioning solution for both of the geodetic receiver and the smartphone, the collected data are processed in differential mode. The broadcast ephemeris is used for differential mode processing, while precise ephemeris obtained from both of the multi GNSS experiment (MGEX) and the new NAVCAST real-time GNSS service are used for post-processing PPP and real-time PPP modes, respectively. Taking the advantage of the availability of dual-frequency observations from the smartphone, undifferenced ionosphere-free (IF) linear combinations of the pseudorange and carrier-phase measurements are formed and used for PPP. Additionally, the extended Kalman filter (EKF) is used as the core algorithm for PPP processing. As the quality of the smartphone’s GNSS observations are found to be relatively low, the carrier-to-noise (C/N) ratio was used as the basis for their weights. It is shown that the dual-frequency GPS/Galileo smartphone is capable of achieving decimeter positioning accuracy in both of the post-processing PPP and real-time PPP modes for the static case, respectively. However, meter-level positioning accuracy is achieved in the kinematic mode.

## 2. GPS/Galileo PPP Mathematical Model

The smartphone’s GPS L1/L5 pseudorange and carrier phase measurements and Galileo E1/E5a counterparts are used. In order to account for the ionospheric delay, un-differenced IF linear combinations of the smartphone’s GPS and Galileo measurements are employed in this research as described in Equations (1) to (4) [13].

(1) $$C_{IF}^{G}\;=\;\;\;\frac{f_{1}^{2}\;}{f_{1}^{2}\;\;-\;\;f_{5}^{2}}\;\;*\;\;C_{1}^{G}\;-\;\;\frac{f_{5}^{2}\;}{f_{1}^{2}\;\;-\;\;f_{5}^{2}}\;\;*\;\;C_{5}^{G}\;\;\;=\;\;\rho^{G}\;\;+\;\;c\;\;*\;\;dt\;{\;}_{r}^{G}\;\;-\;\;\;c\;\;*\;\;dt\;{\;}_{s}^{G}\;+\;\;T^{G}\;\;+\;\;\varepsilon \;{\;}_{CIF}^{G}$$

(2) $$\Phi_{IF}^{G}\;=\;\;\;\frac{f_{1}^{2}\;}{f_{1}^{2}\;\;-\;\;f_{5}^{2}}\;\;*\;\;\Phi_{1}^{G}\;-\;\;\frac{f_{5}^{2}\;}{f_{1}^{2}\;\;-\;\;f_{5}^{2}}\;\;*\;\;\Phi_{5}^{G}\;\;\;=\;\;\rho^{G}\;\;+\;\;c\;\;*\;\;dt\;{\;}_{r}^{G}\;\;-\;\;\;c\;\;*\;\;dt\;{\;}_{s}^{G}\;+\;\;T^{G}\;\;+\;\;N\;{\;}_{IF}^{G}+\;\;\varepsilon \;{\;}_{\Phi IF}^{G}$$

(3) $$C_{IF}^{E}\;=\;\;\;\frac{f_{1}^{2}\;}{f_{1}^{2}\;\;-\;\;f_{5}^{2}}\;\;*\;\;C_{1}^{E}\;-\;\;\frac{f_{5}^{2}\;}{f_{1}^{2}\;\;-\;\;f_{5}^{2}}\;\;*\;\;C_{5}^{E}\;\;\;\;=\;\;\rho^{E}\;\;+\;\;c\;\;*\;\;dt\;{\;}_{r}^{G}\;\;-\;\;\;c\;\;*\;\;dt\;{\;}_{s}^{E}\;+\;\;T^{E}\;\;+ISB+\;\;\varepsilon \;{\;}_{CIF}^{E}$$

(4) $$\Phi_{IF}^{E}\;=\;\;\;\frac{f_{1}^{2}\;}{f_{1}^{2}\;\;-\;\;f_{5}^{2}}\;\;*\;\;\Phi_{1}^{E}\;-\;\;\frac{f_{5}^{2}\;}{f_{1}^{2}\;\;-\;\;f_{5}^{2}}\;\;*\;\;\Phi_{5}^{E}\;\;\;=\;\;\rho^{E}\;\;+\;\;c\;\;*\;\;dt\;{\;}_{r}^{G}\;\;-\;\;\;c\;\;*\;\;dt\;{\;}_{s}^{E}\;+\;\;T^{E}\;\;+N\;{\;}_{IF}^{E}+\;\;ISB+\;\;\varepsilon \;{\;}_{\Phi IF}^{E}$$

where, *G* and *E* refer to GPS and Galileo respectively; *C1* and *C5* refer to the C/A pseudorange measurements on L1/ L5 for GPS and on E1/E5a for Galileo; *Φ_1_* and *Φ_5_* refer to the carrier phase measurements on L1/ L5 for GPS and on E1/E5a for Galileo; *f_1_* and *f_5_* are the L1/E1 and L5/E5a frequencies; *ρ^G^*, *ρ^E^* are the geometric range between the receiver and corresponding satellite; *c* is the speed of light; ${dt}_{r}^{G}$ is the GPS receiver clock error, which includes the GPS IF linear combination of receiver code hardware delay; ${dt}_{s}^{G}$/${dt}_{s}^{E}$ are the GPS/Galileo satellite clock errors, which include the GPS/Galileo IF linear combination of satellite code hardware delay; *T^G^* and *T^E^* are the total tropospheric delay for the GPS and Galileo, respectively; $N_{IF}^{G}$/$N_{IF}^{E}$ are the GPS/Galileo non-integer IF ambiguity terms, which are a combination of the GPS/Galileo IF linear combinations of satellite code hardware delay, satellite phase hardware delay, receiver code hardware delay, and receiver phase hardware delay; *ISB* refers to the inter-system bias between the Galileo and GPS satellite systems, which equal the difference between the GPS and Galileo IF linear combination of satellite code hardware delay; *ε* refers to the multipath effect and un-modeled errors.

GPS satellites clock correction includes IF linear combination of satellites code hardware delays on L1/L2 and Galileo satellites clock correction includes IF linear combination of satellites code hardware delays on E1/E5a [14]. In order to be consistent with GPS satellites clock correction, GPS IF linear combination of smartphone’s pseudorange measurements are corrected by *DCB_corr_*.

(5) $$DCB\;{\;}_{corr}\;\;\;=\left(1.546*DCB\;{\;}_{P1-P2}\;\;+\;\;DCB\;{\;}_{P1-C1}\;\;-\;1.26\;DCB_{C1-C5}\right)$$

where, *DBC_corr_*, is differential code bias (DCB) correction; *DBC_P1−P2_*, *DBC_P1−C1_*, *DBC_C1−C5_* are daily GPS DCBs that can be extracted from the bias solution independent exchange format (Bias-SINEX) file.

Additionally, the zenith dry component of the tropospheric delay is accounted for using the Saastamoinen model [15]. Dry and wet mapping functions are determined using Vienna mapping function (VMF), which is based on coefficients derived from the global pressure and ‘temperature 2 wet’ (GPT2w) model [16]. The effects of relativity, sagnac delay, phase centre offset and variation, Earth tides, ocean loading, and phase wind up are modeled as described in [17]. The IF linear combination of pseudorange and carrier phase measurements after accounting for all mentioned errors for GPS and Galileo are summarized in Equations (6) to (9).

(6) $${\tilde{C}}_{IF}^{G}\;=\;\;\;\rho^{G}\;\;+\;\;b\;{\;}_{r}^{G}\;\;+\;\;m_{w}^{G}\;\;*\;\;zwd\;{\;}^{G}\;+\;\;\;\;\varepsilon \;{\;}_{CIF}^{G}$$

(7) $${\tilde{\Phi }}_{IF}^{G}\;=\;\;\;\rho^{G}\;\;+\;\;b\;{\;}_{r}^{G}\;\;+\;\;m_{w}^{G}\;\;*\;\;zwd\;{\;}^{G}\;+\;\;N\;{\;}_{IF}^{G}+\;\;\varepsilon \;{\;}_{CIF}^{G}$$

(8) $${\tilde{C}}_{IF}^{E}\;=\;\;\;\rho^{E}\;\;+\;\;b\;{\;}_{r}^{G}\;\;+\;\;m_{w}^{E}\;\;*\;\;zwd\;{\;}^{E}\;+ISB+\;\;\;\;\varepsilon \;{\;}_{CIF}^{E}$$

(9) $${\tilde{\Phi }}_{IF}^{E}\;=\;\;\;\rho^{E}\;\;+\;\;b\;{\;}_{r}^{G}\;\;+\;\;m_{w}^{E}\;\;*\;\;zwd\;{\;}^{E}\;+ISB+\;\;N\;{\;}_{IF}^{E}+\;\;\varepsilon \;{\;}_{CIF}^{E}$$

where, *zwd* is the zenith wet delay; *m_w_* is the wet mapping function; $b_{r}^{G}=c*{\mathrm{dt}}_{r}^{G}$.

## 3. Extended Kalman Filter Mathematical Models and Implementation

The EKF is employed in this research as the core algorithm for the estimation process. It can be implemented in two consequent stages, namely prediction and update [18]. The prediction stage is summarized in Equations (10) to (11).

(10) $$x\;{\;}_{k\;}(-)\;\;=\;\;\;\Phi \;{\;}_{k\;,\;\;k-1}\;\;*\;\;\;x\;{\;}_{k-1}$$

(11) $$P\;{\;}_{k\;}(-)\;\;=\;\;\;\Phi \;{\;}_{k\;,\;\;k-1}\;\;*\;\;\;P\;{\;}_{k-1}(+)\;\;\;*\;\;\Phi_{k\;\;,\;\;k-1}^{T}\;\;+\;\;\;Q\;{\;}_{k\;\;,\;\;k-1}$$

where, (-) refers to the predicted state; (+) refers to the updated state; *k* and *k-1* are two following epochs; *x* is the state vector which includes position, receiver clock error, zenith wet tropospheric delay, ISB, and ambiguity parameters; *P* is the state vector covariance matrix with initial variances as described in reference [19]; Φ is the transition matrix which is the identity matrix; *Q* is the covariance matrix of the process noise in which the static positon and ambiguity are modeled as constants, while receiver clock error, zenith wet delay, and ISB are modeled as random walk process [20]. The new pseudorange and carrier phase measurements are used to update the predicted state as in Equations (12) to (14).

(12) $$K\;{\;}_{k}\;=\;\;\;P\;{\;}_{k\;}(-)\;\;\;*\;\;\;H\;{\;}_{k}^{T}\;\;*\;\;\left[H{\;}_{k}\;\;*\;\;\;P\;{\;}_{k\;}(-)\;\;\;\;*\;\;\;H\;{\;}_{k}^{T}\;\;+\;\;R\;{\;}_{k}\right]\;{\;}^{-1}$$

(13) $$P\;{\;}_{k\;}(+)\;\;=\;\;\;\;\;\;\left(\;I\;\;-\;\;K\;{\;}_{k}\;\;H\;{\;}_{k\;}\;\right)\;\;P\;{\;}_{k\;}(-)\;$$

(14) $$x\;{\;}_{k\;}(+)\;\;=\;\;\;\;x\;{\;}_{k\;}(-)\;\;\;+\;\;\;K\;{\;}_{k}\;\;\left[y_{k}\;-\;\;h\;\;\;\;\;\left(x\;{\;}_{k}\;\;\left(-\right)\;\;\right)\right]$$

where, *K* is the Kalman gain; *H* is the matrix of partial derivatives with respect to the unknowns; *R_k_* is the measurements covariance matrix, which is a diagonal matrix with the pseudorange and carrier phase measurements variances as the diagonal elements. Due to the low carrier to noise (C/N) ratio of smartphone’s measurements, the variances can be modeled as a function of C/N as in reference [21]; *y* includes the pseudorange and carrier phase measurements as in Equation (15).

(15) $$y\;\;=\;\;\left[\begin{array}{ccc} {\tilde{C}}_{IF}^{1} & .. & {\tilde{C}}_{IF}^{n}\;\begin{array}{ccc} {\tilde{C}}_{IF}^{1} & .. & {\tilde{C}}_{IF}^{m}\;\;\begin{array}{ccc} {\tilde{\Phi }}_{IF}^{1} & .. & {\tilde{\Phi }}_{IF}^{n}\;\begin{array}{ccc} {\tilde{\Phi }}_{IF}^{1} & .. & {\tilde{\Phi }}_{IF}^{m} \end{array} \end{array} \end{array} \end{array}\right]\;{\;}^{T}$$

where, *n* and *m* refer to GPS and Galileo satellites numbers; h is the measurement vector.

(16) $$h\;\;=\left[\begin{array}{cccc} A & B & C & D \end{array}\right]\;{\;}^{T}$$

where,

(17) $$A=\left[\begin{array}{cccc} \rho^{1,G}\;\;+\;\;b\;{\;}_{r}^{G}\;\;+\;\;m_{w}^{1,G}\;\;*\;\;zwd\;\;\; & . & . & \rho^{n,G}\;\;+\;\;b\;{\;}_{r}^{G}\;\;+\;\;m_{w}^{n,G}\;\;*\;\;zwd\;\;\; \end{array}\right]\;{\;}^{T}$$

(18) $$B=\left[\begin{array}{cccc} \rho^{1,E}\;\;+\;\;b\;{\;}_{r}^{G}\;\;+\;\;ISB+m_{w}^{1,E}\;\;*\;\;zwd\;\;\;\;\; & . & . & \rho^{1,E}\;\;+\;\;b\;{\;}_{r}^{G}\;\;+\;\;ISB+m_{w}^{m,E}\;\;*\;\;zwd\;\;\;\;\; \end{array}\right]\;{\;}^{T}$$

(19) $$C=\left[\begin{array}{cccc} \rho^{1,G}\;\;+\;\;b\;{\;}_{r}^{G}\;\;+\;\;m_{w}^{,G1}\;\;*\;\;zwd\;+N\;{\;}_{IF}^{1,G}\;\; & . & . & \rho^{n,G}\;\;+\;\;b\;{\;}_{r}^{G}\;\;+\;\;m_{w}^{n,G}\;\;*\;\;zwd+N\;{\;}_{IF}^{n,G}\;\;\; \end{array}\right]\;{\;}^{T}$$

(20) $$D=\left[\begin{array}{cccc} \rho^{1,E}\;\;+\;\;b\;{\;}_{r}^{G}\;\;+\;\;ISB+m_{w}^{1,E}\;\;*\;\;zwd+N\;{\;}_{IF}^{1,E}\;\;\;\;\; & . & & \rho^{1,E}\;\;+\;\;b\;{\;}_{r}^{G}\;\;+\;\;ISB+m_{w}^{m,E}\;\;*\;\;zwd+N\;{\;}_{IF}^{m,E}\;\;\;\;\; \end{array}\right]\;{\;}^{T}$$

## 4. Data Collection

Two Trimble R9 geodetic receivers with the capability of tracking GPS and Galileo satellites were used in this research. GPS L1/L2 and Galileo E1/E5a pseudorange and carrier phase measurements were collected from the geodetic receivers. GNSS raw measurements from the smartphone can be produced in Rinex 3.03 format using the “Rinex ON” application, which has been developed by Nottingham Scientific Limited (NSL) as part of the Flamingo project [22]. The smartphone can receive only L1/L5 signals for GPS and E1/E5a for Galileo. The smartphone’s GPS L1/L5 and Galileo E1/E5a pseudorange and carrier phase measurements were collected as presented in Table 1. 

**Figure 1:**
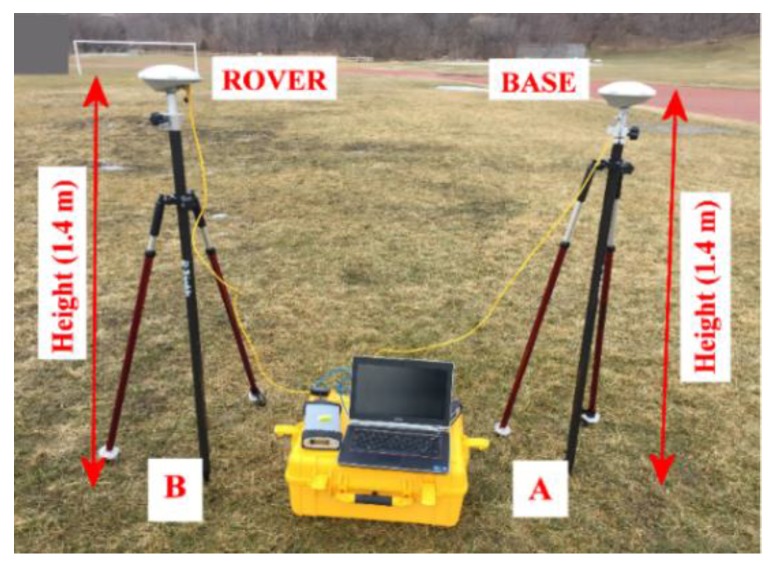
Setup of Trimble R9 geodetic receivers.

**Figure 2:**
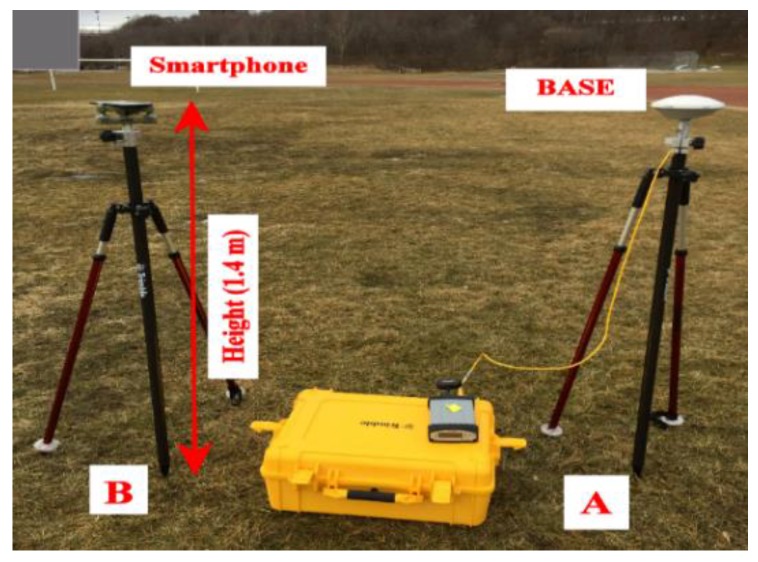
Setup of Trimble R9 geodetic receiver and smartphone.

As shown in Figure 1, one of the geodetic receivers was set over point A, which was the base station, and the other one was set over point B, which was the rover station, with a baseline of 1.30 m. As shown in Figure 2, the rover geodetic receiver was replaced with the smartphone placed horizontally on its back side using a surveying tribrach. Datasets from both of the geodetic receivers and the smartphone were collected in static mode at the Riverdale Park, Toronto, Ontario, Canada under an open sky environment. Three hours of static data from the base receiver were collected. Additionally, about one hour of data from each of the rover geodetic receiver and the smartphone were collected in order to be processed in the DGNSS mode and to ensure that these data were collected under the same environment. The same procedure was repeated for the second dataset collection.

**Figure 3:**
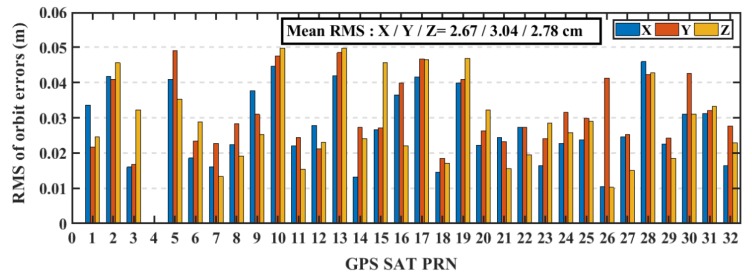
RMS of GPS satellites’ orbits based on NAVCAST real-time products.

**Figure 4:**
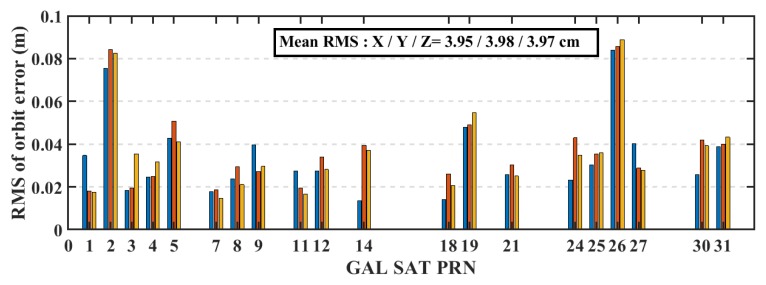
RMS of Galileo satellites’ orbits based on NAVCAST real-time products.

**Figure 5:**
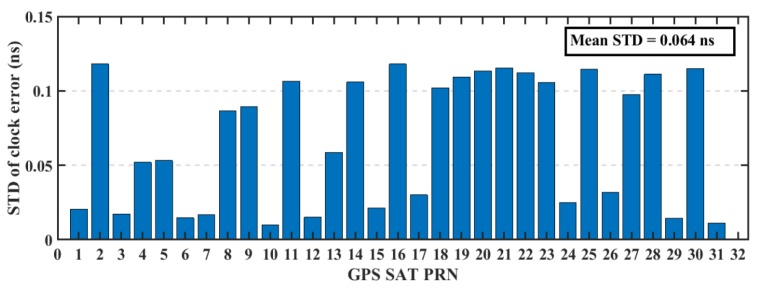
STD of GPS satellites’ clocks based on NAVCAST real-time products.

**Figure 6:**
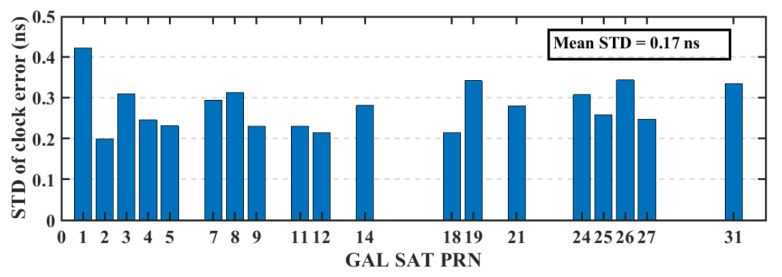
STD of Galileo satellites’ clocks based on NAVCAST real-time products.

The real-time precise ephemeris obtained from the NAVCAST GNSS PPP service were used in real-time PPP mode. It was announced on October 29, 2018 by Spaceopal Company in Germany [23] and it provides GPS and Galileo orbit and clock corrections to be used with the broadcast ephemeris in order to convert it to the precise ephemeris counterpart. These products are produced based on the RETICLE algorithm developed by the German Aerospace Centre (DLR) [24,25]. NAVCAST GPS/Galileo orbit and clock corrections that are broadcasted every 30 and 5 s, respectively. The NAVCAST real-time products are evaluated in this research by using the precise ephemeris form the MGEX as a reference. The differences between NAVCAST products and precise ephemeris are analyzed over two consecutive days (DOY 36 and 37 of the year 2019). The resulted clock differences are aligned to a reference satellite in order to remove any systematic biases [26]. In this research, GPS satellite PRN 32 and Galileo satellite PRN number 30 are chosen as reference satellites. For GPS satellites, the orbit accuracy is about 2.35 cm, 2.51 cm, 2.27 cm in x, y, and z directions with RMS of 2.67 cm, 3.04 cm, and 2.78 cm as presented in Figure 3. For Galileo satellites, the orbit accuracy is about 2.95 cm, 3.42 cm, 3.38 cm in x, y, and z directions with RMS of 3.95 cm, 3.98 cm, and 3.97 cm for x, y, and z directions as presented in Figure 4. The STD values of GPS satellites clock errors are generally smaller than 0.15 ns with mean STD of 0.064 ns, which is an indication of the accurate clock products, as shown in Figure 5. The STD values of Galileo satellites clock errors are smaller than 0.4 ns with mean RMS of 0.17 ns, as shown in Figure 6.

## 5. Smartphone’s Data Quality

**Figure 7:**
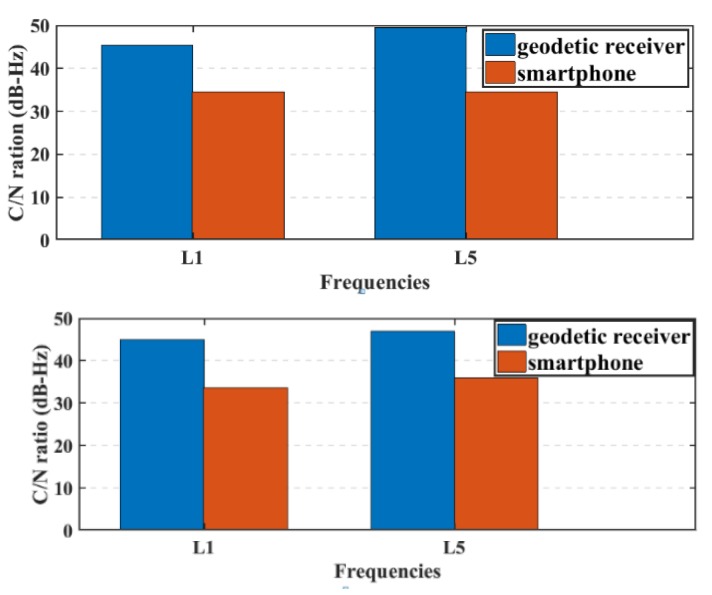
Average C/N for GPS and Galileo satellites, respectively.

The transmitted GNSS signals have right-handed circular polarization. As a result, the optimum choice of GNSS antenna is to be right-handed circularly polarized (RHCP) due to the fact that it can discriminate between direct GNSS signal and indirect GNSS signal, which is transformed to left-handed circularly polarized (LHCP) signal [27]. The embedded antennas in smartphones are linear-polarized antennas, which cannot discriminate between direct (RHCP) and indirect (LHCP) signals, leading to noisy measurements and lower carrier-to-noise (C/N) ratio [6]. The quality of the smartphone measurements are lower than the geodetic receiver counterpart, which is attributed to the quality of the receiver-antenna combination. The average C/N values obtained from the smartphone are low with about 10 dB-Hz compared to the geodetic receiver counterpart, as presented in Figure 7.

**Figure 8:**
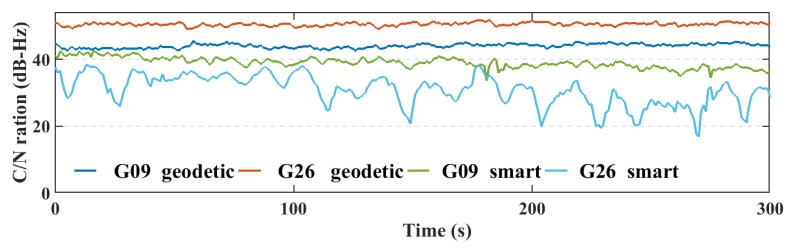
L1 C/N values from the geodetic receivers (geodetic) and the smartphone (smart) for GPS.

**Figure 9:**
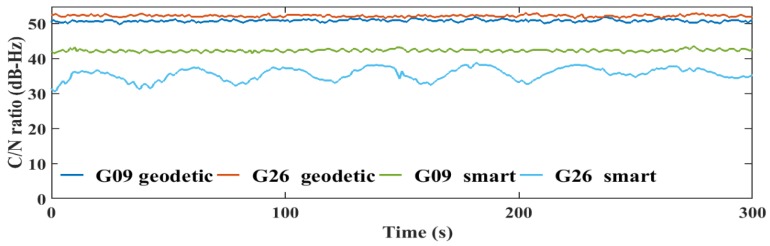
L5 C/N values from the geodetic receivers (geodetic) and the smartphone (smart) for GPS.

**Figure 10:**
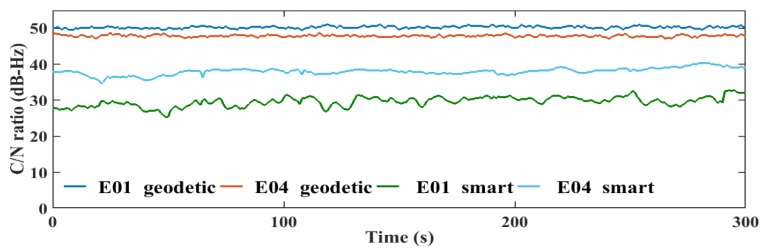
L1 C/N values from the geodetic receiver (geodetic) and the smartphone (smart) for Galileo.

**Figure 11:**
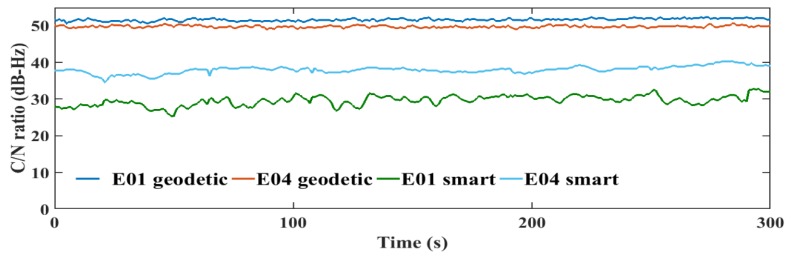
L5 C/N values from the geodetic receiver (geodetic) and the smartphone (smart) for Galileo.

Additionally, the C/N values from the smartphone are highly variable, which is another indicator of the smartphone’s low quality measurements, as presented in Figure 8, Figure 9, Figure 10 and Figure 11.

## 6. Results and Analysis

### 6.1. GNSS Differential Solution 

GPS/Galileo dual-frequency observations from both of the smartphone and rover geodetic receiver are processed in differential mode using the TBC software, due to its ability to process multi-constellation multi-frequency observations. The broadcast ephemeris for GPS and Galileo are used to account for satellite orbit and clock errors. The nearest CORS station to the data collection site is Port Weller (PWEL) station with about 47 km baseline. Unfortunately, the available observations from PWEL include only GPS L1/L2 measurements, which makes the use of PW EL as the base station is not applicable, since the smartphone provides only GPS L1/L5 observations. On the other hand, the Trimble R9 receiver can provide L1/L2/L5 measurements for GPS and E1/E5a measurements for Galileo. So, three hours of GNSS observations from the base geodetic receiver are uploaded to Natural Resource Canada (NRCAN) online PPP tool to determine the coordinates of the base station. The DGNSS solutions for the first dataset, which represent the final solutions for both of the geodetic receiver and smartphone, are presented in Table 2.

### 6.2. PPP Positioning Performance Using the Smartphone

**Figure 12:**
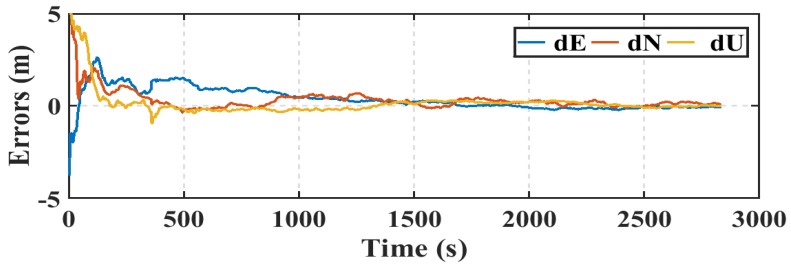
Smartphone PPP solution errors in East, North, and Up directions (dE, dN, dU).

**Figure 13:**
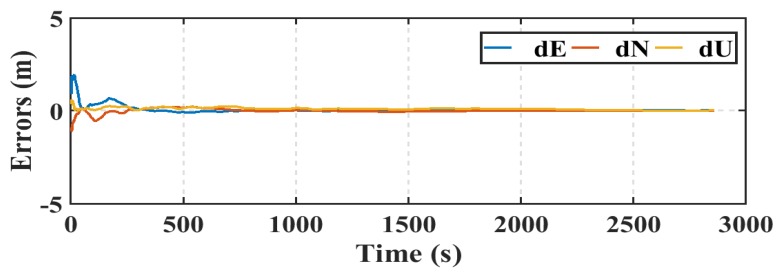
Geodetic receiver PPP solution errors in East, North, and Up directions (dE, dN, dU).

The GPS/Galileo observations from both of the smartphone and rover geodetic receiver are processed using the PPP software developed at Ryerson University as described in Section 1 and Section 3. Precise ephemeris from the MGEX, initial state vector covariance matrix, and initial noise covariance matrix as described in reference [19] are used. However, the process noise variances for the position component are set to zeros because the processing mode is static. Since the exact location of the smartphone antenna is unknown, both of the smartphone and geodetic receiver PPP solutions accuracy are assessed compared to their DGNSS counterparts. PPP solution errors in East, North, and Up directions for both of the smartphone and the geodetic receiver are presented in Figure 12 and Figure 13, respectively. 

As in Table 3, the smartphone’s PPP solution RMS errors are 0.507 m, 0.473 m, and 0.559 m in East, North, and Up directions compared to 0.208 m, 0.109 m, and 0.117 m for the geodetic receiver counterpart. The means of the positioning errors are 0.061, 0.056, and 0.100 m in East, North, and Up directions for geodetic receiver, compared to 0.364, 0.310, and 0.305 m for the smartphone’s counterpart. 

**Figure 14:**
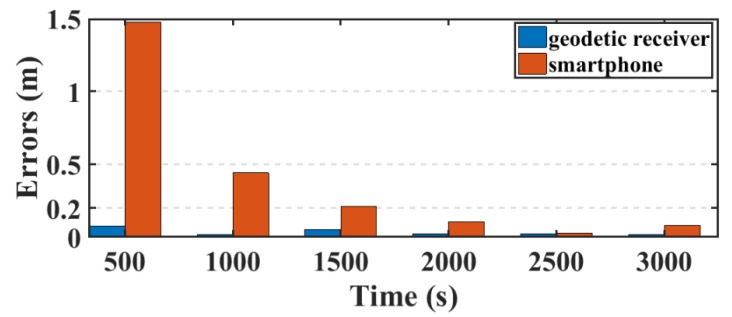
Position errors in East direction (meters).

**Figure 15:**
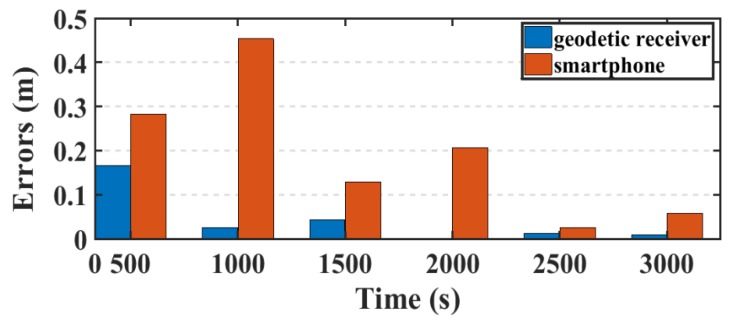
Position errors in North direction (meters).

**Figure 16:**
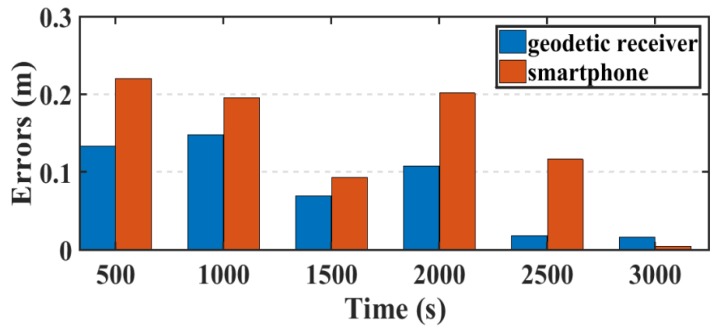
Position errors in Up direction (meters).

Another parameter used in this research to assess the smartphone’s PPP solution, is its convergence behavior compared with the geodetic receiver’s PPP solution counterpart. Figure 14, Figure 15, and Figure 16 show the positioning errors at specific time tags for both of the PPP solutions. For the geodetic receiver’s PPP solution, about 8 minutes are needed to achieve less than 0.2 m accuracy for the East component compared to about 25 min for the smartphone’s PPP solution to achieve the same level of accuracy. Additionally, about 8 min are required for the smartphone’s PPP solution to achieve less than 0.3 m accuracy for the North component, compared to less than 0.2 m for the geodetic receiver counterpart. Moreover, about 8 min are needed to reach 0.2 m accuracy for the Up component for the smartphone’s PPP solution and about 0.1 m for the geodetic receiver counterpart. 

### 6.3. Smartphone Real-time PPP Solution

**Figure 17:**
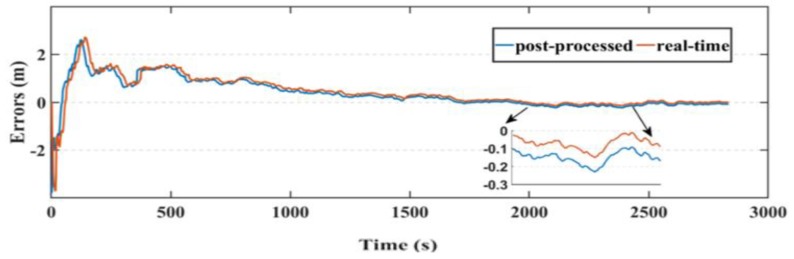
Position errors in East direction (first dataset).

**Figure 18:**
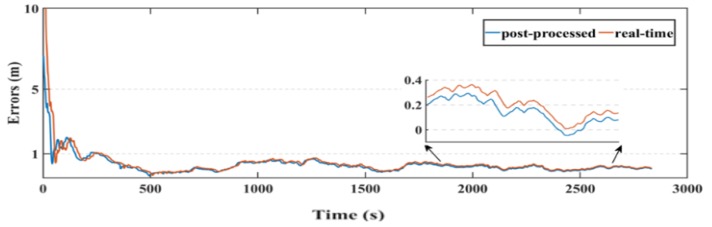
Position errors in North direction (first dataset).

**Figure 19:**
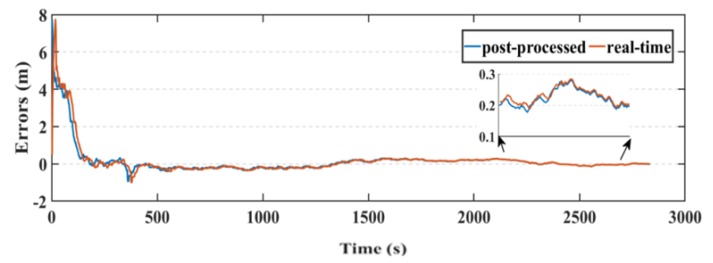
Position errors in Up direction (first dataset).

The PPP solution is assessed in both of static and kinematic positioning modes. The real-time PPP processing is performed using the precise ephemeris resulted from the NAVACAT products. The resulted static real-time PPP (RT-PPP) positioning solution, using NAVCAST products, and the post-processed PPP solution of the first data set are presented in Figure 17, Figure 18, and Figure 19. 

The RMS of the post-processed PPP solution errors are 0.507 m, 0.473 m, 0.559 m in East, North, and Up directions, respectively, compared to 0.558 m, 0.542 m, and 0.621 m for the RT-PPP counterpart as in Table 4.

**Figure 20:**
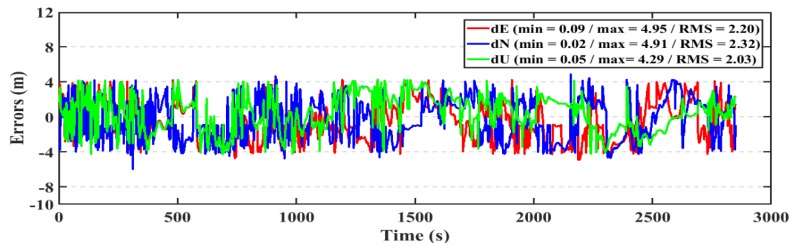
Position errors for the post-processed PPP solution (kinematic/first dataset).

**Figure 21:**
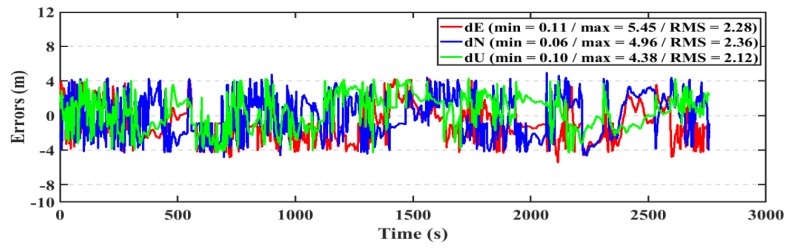
Position errors for the RT-PPP solution (kinematic/first dataset).

For the kinematic test, the post-processed PPP and RT-PPP positioning solutions for the first dataset are presented in Figure 20, Figure 21, respectively. The RMS of the post-processed PPP solution errors are 2.20 m, 2.32 m, 2.03 m in East, North, and Up directions, respectively, compared to 2.28 m, 2.36 m, and 2.12 m for the RT-PPP counterpart. 

**Figure 22:**
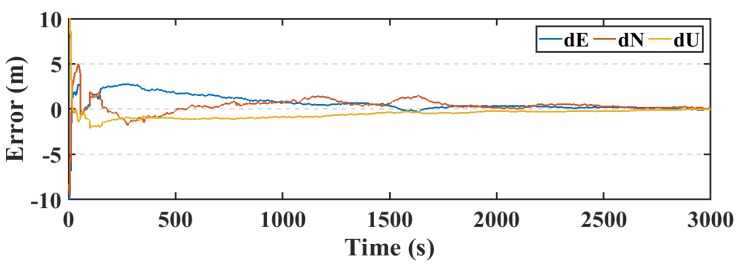
Position errors for the post-processed PPP solution (static/second dataset).

**Figure 23:**
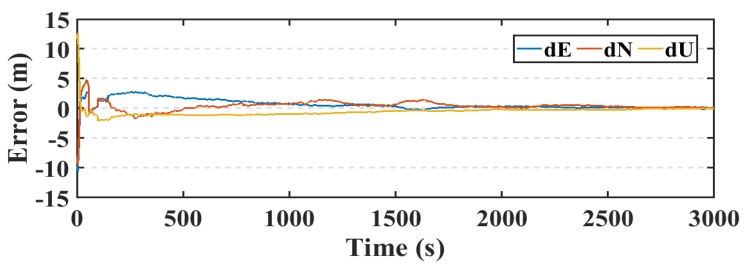
Position errors for the RT-PPP solution (static/second dataset).

The resulted static real-time PPP (RT-PPP) positioning solution, using NAVCAST products, and the post-processed PPP solution for the second data set are presented in Figure 22 and Figure 23. The RMS of the post-processed PPP solution errors are 0.668 m, 0.614 m, 0.589 m in East, North, and Up directions, respectively, compared to 0.844 m, 0.647 m, and 0.661 m for the RT-PPP counterpart as in Table 5.

**Figure 24:**
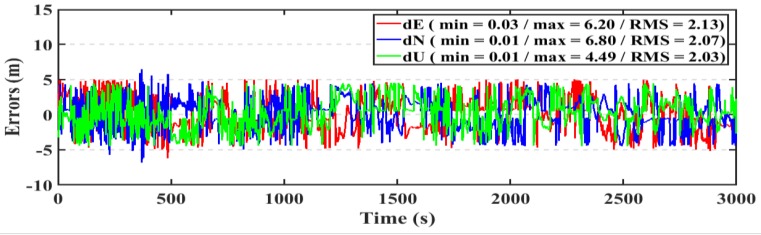
Position errors for the post-processed PPP solution (kinematic/second dataset).

**Figure 25:**
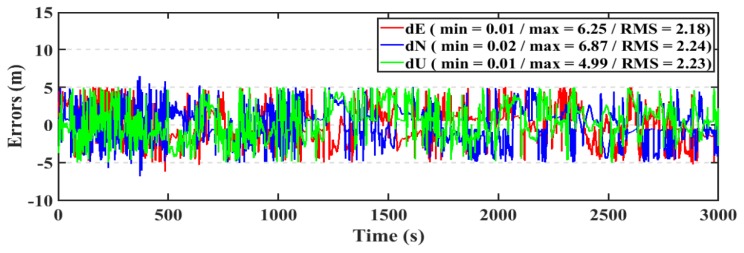
Position errors for the RT-PPP solution (kinematic/second dataset).

For the kinematic test, the post-processed PPP and RT-PPP positioning solutions of the second dataset are presented in Figure 24, Figure 25, respectively. The RMS of the post-processed PPP solution errors are 2.13 m, 2.07 m, 2.03 m in East, North, and Up directions, respectively, compared to 2.18 m, 2.24 m, and 2.23 m for the RT-PPP counterpart. 

## 7. Conclusions

The positioning performance of the new dual-frequency GNSS smartphone, Xiaomi mi 8, was assessed in both of post-processing and real-time PPP positioning modes. The analysis of the C/N ratio of the smartphone’s GNSS observations showed that it is lower than its geodetic receiver’s counterpart by about 10 dB-Hz. Ryerson University’s PPP software was used to process the smartphone’s dual-frequency GPS/Galileo observations. It was shown that, using the C/N ratio of the smartphone’s observations as the basis for their weights, its PPP solution achieved decimeter-level positioning accuracy in post-processing mode. The smartphone’s real-time PPP was assessed by employing the new NAVCAST real-time GNSS services and its real-time PPP solution showed decimeter-level positioning accuracy. The smartphone showed meter-level kinematic positioning accuracy in both of the real-time and post-processed modes. 

## Figures and Tables

**Table 1:** Data collected.

Receiver Type	GPS	Galileo
Trimble R9	C1C and L1C, C2W and L2W	C1C and L1C, C5X and L5X
smartphone	C1C and L1C, C5X and L5X	C1C and L1C, C5X and L5X

**Table 2:** Geodetic receiver and smartphone DGNSS solutions.

Receiver Type	DGNSS ITRF Solution (m)		
	X	Y	Z
Geodetic	85,3516.578	-454,1447.832	438,1617.828
Smartphone	85,3516.507	-454,1447.881	438,1617.659

**Table 3:** Mean and RMS for East, North, and Up components.

Receiver Type	dE (m)		dN (m)		dU (m)	
	Mean	RMS	Mean	RMS	Mean	RMS
Geodetic	0.061	0.208	0.056	0.109	0.100	0.117
Smartphone	0.364	0.507	0.310	0.473	0.305	0.559

**Table 4:** Mean and RMS of East, North, and Up errors for the first data set.

PPP Solution	dE (m)		dN (m)		dU (m)	
	Mean	RMS	Mean	RMS	Mean	RMS
post-processed	0.364	0.507	0.310	0.473	0.305	0.559
real-time	0.430	0.558	0.429	0.542	0.424	0.621

**Table 5:** Mean and RMS of East, North, and Up errors for the second data set.

PPP Solution	dE (m)		dN (m)		dU (m)	
	Mean	RMS	Mean	RMS	Mean	RMS
post-processed	0.582	0.668	0.325	0.614	0.466	0.589
real-time	0.650	0.844	0.390	0.647	0.555	0.661
